# Transitioning from the safety of the womb to the outside world for neonates with life-threatening cardiovascular conditions: The *IMmediate Postpartum Access to Cardiac Therapy* (IMPACT) Procedure

**DOI:** 10.1007/s00246-025-03775-y

**Published:** 2025-01-20

**Authors:** Asif Padiyath, Jennifer M. Lynch, Lisa M. Montenegro, Susan C. Nicolson, Olivia Nelson, Anita L. Szwast, Amanda J. Shillingford, Christine B. Falkensammer, Jill J. Savla, Julie Moldenhauer, Nahla Khalek, Jack Rychik

**Affiliations:** 1 Division of Cardiothoracic Anesthesiology, Department of Anesthesiology and Critical Care Medicine, Children’s Hospital of Philadelphia, Philadelphia, PA USA; 2 Division of Cardiology, Department of Pediatrics, Children’s Hospital of Philadelphia, Philadelphia, PA USA; 3 Department of Anesthesiology and Critical Care, Perelman School of Medicine at the, University of Pennsylvania, Philadelphia, PA USA; 4 Department of Bioengineering, School of Engineering and Applied Science, University of Pennsylvania, Philadelphia, PA USA; 5 Perelman School of Medicine at the University of Pennsylvania, University of Pennsylvania Health System, Philadelphia, PA USA; 6 Section of Fetal Anesthesia, Division of General Anesthesiology, Department of Anesthesiology and Critical Care Medicine, Children’s Hospital of Philadelphia, Philadelphia, PA USA; 7 Fetal Heart Program, Division of Cardiology. Department of Pediatrics, Children’s Hospital of Philadelphia, Philadelphia, PA USA; 8 Department of Pediatrics, Perelman School of Medicine at the University of Pennsylvania, Philadelphia, PA USA; 9 Division of Maternal-Fetal Medicine, Richard D. Wood Jr. Center for Fetal Diagnosis and Treatment (CFDT), The Children’s Hospital of Philadelphia, Philadelphia, PA USA; 10 Department of Surgery, Perelman School of Medicine at the, University of Pennsylvania, Philadelphia, PA USA; 11 Department of Obstetrics and Gynecology, Perelman School of Medicine at the University of Pennsylvania, Philadelphia, PA USA; 12 Fontan Rehabilitation, Wellness, Activity and Resilience Development (FORWARD) Program, Division of Cardiology, Department of Pediatrics, Children’s Hospital of Philadelphia, Philadelphia, PA USA

**Keywords:** IMPACT, Fetal cardiology, Multidisciplinary, Transposition of great arteries, Hypoplastic left heart syndrome, Intervention, Access, Immediate, Cesarean delivery

## Abstract

The IMmediate Postpartum Access to Cardiac Therapy (IMPACT) procedure is a multidisciplinary, collaborative, highly coordinated clinical service in which a planned delivery and intensive neonatal care are offered for conditions where there is a high likelihood of postnatal instability. This process includes prenatal consultation with the parent(s), involving each service engaged with the delivery, postnatal resuscitation, and procedural care. A Cesarean section delivery is planned in an operating room with immediate access to a multifunctional procedural suite where the neonate can undergo rapid cardiac evaluation and initiation of interventional treatments which can have a positive, life-saving impact**.** This review describes the details of this unique procedure and multidisciplinary program at one institution.

## Introduction

Advancements in fetal echocardiography over the past several decades have allowed clinicians to prenatally identify more than 50% of fetuses with critical congenital heart diseases (CCHD) [1–4]. Improved understanding of the fetal-to-newborn change in physiology that occurs with CCHD has allowed clinicians to identify fetuses who at the time of separation from maternal-placental support, manifest immediate life-threatening findings that may benefit from urgent interventions [5]. Candidates include fetuses with a variety of diagnoses such as hypoplastic left heart syndrome (HLHS) with intact or restrictive atrial septum [6, 7], d-transposition of the great arteries with intact ventricular septum and restrictive atrial septum (D-TGA/IVS) [8–11], congenital complete heart block with anticipated hemodynamic instability [12, 13], total anomalous pulmonary venous return with obstruction, and a host of other cardiac anomalies. Any delay in providing medical, surgical, or catheter-based interventional care to these neonates with CCHD within the first few minutes to hours of life can potentially result in significant morbidity or mortality [14].

Given the time-sensitive nature of the care for this subset of neonates including careful newborn assessment, acquisition of vascular access, airway and ventilatory management, and potential cardiovascular interventions, a specially designed system with multidisciplinary capacities is required to provide optimal care. Proper facilities and a highly skilled team of providers can best provide the opportunity to achieve a successful transition from the safety of the womb to the outside world. The proximity of the delivery room to the location where immediate postnatal care takes place is critical to providing this optimal care. Close to 50% of the pediatric cardiac programs that offer cardiac surgical care for neonates in the United States are located in a free-standing children’s hospitals [15]. The logistics of transporting neonates needing immediate postnatal intervention from the delivery room to the cardiac care area could pose significant challenges. Many of these cardiac centers may have their obstetric services in a different location separated from the pediatric cardiac care area, with numerous logistical obstacles that can delay urgent, life-saving care.

Recognizing the desire to offer an individualized and seamlessly coordinated plan of care for these fragile newborns and to offer the best opportunity for optimal outcomes for complex conditions, we have adopted a multidisciplinary approach—The IMmediate Postpartum Access to Cardiac Therapy (IMPACT) strategy of care [16, 17]. The objective of the IMPACT procedure is to provide immediate postnatal care to those who would otherwise be at high risk of adverse outcomes if not urgently treated in this manner. The strategy involves careful orchestration of services to include maternal care with a scheduled Cesarean delivery (CD) delivery adjacent to a venue that can provide immediate newborn evaluation and an opportunity for planned urgent intervention(s). In this review, we describe the inception of the concept, patient selection, multidisciplinary team approach, and the planning and coordination involved.

### Conceptual Development of the IMPACT Procedure

In 2008, The Children’s Hospital of Philadelphia (CHOP) was the first freestanding children’s hospital in North America to open an onsite delivery unit offering full scale obstetrical services with 24/7 coverage [18]. Delivering newborns with congenital heart disease at a children’s hospital prompted the design of a system of stratification for communication of delivery care needs for the neonate. A four-tiered system of delivery room care for cardiovascular conditions was developed, based on the estimated needs of resources and personnel expertise necessary to stabilize the newborn. (Table 1). Neonates with Class I conditions are expected to be hemodynamically stable after delivery, receive standard neonatal resuscitation, and the ductus arteriosus is allowed to close naturally. Class I can be further stratified into conditions that do not benefit from arterial line monitoring (Class IA) and conditions in which prostaglandin-E (PGE) infusion for ductal patency is not desired, but the transition from fetal to newborn physiology with ductal closure benefits from close monitoring, hence arterial blood pressure monitoring via an umbilical arterial line is indicated (Class IB).

**Table 1:** Severity classification for delivery of fetuses with cardiovascular disease at The Children’s Hospital of Philadelphia—Special Delivery Unit

	Definition	PGE?	Action	Access	Example anomalies/diseases*
CLASS Ia	Simple cardiovascular anomaly or disease, requiring delivery in the SDU but no hemodynamic instability anticipated at delivery	NO	Neonatology to manage delivery	PIV, no UAC	• LV/RV size discrepancy with suspicion of possible Coarctation of the aorta • AV Canal, balanced • TOF with mild PS (“Pink TOF”) • Benign arrhythmia (non-sustained SVT, no hydrops, no ventricular dysfunction)
CLASS Ib			Neonatology to manage delivery	UVC + UAC	• Truncus Arteriosus • LV-RV disproportion, with intent to test ductal closure for possible coarctation of aorta
CLASS II	Cardiovascular anomaly or disease of moderate severity, including ductal dependent lesions. Hemodynamic instability is possible, but unlikely and not anticipated at delivery	YES	Neonatology to manage delivery	UVC + UAC	• HLHS, no risk factors (open inter-atrial communication, no significant TR, good RV function) • Single ventricle with critical (suspect ductal dependent) pulmonary outflow obstruction (e.g., tricuspid atresia) • Single ventricle with critical (suspect ductal dependent) systemic outflow obstruction (e.g., Double-inlet LV with arch obstruction) • Coarctation of the aorta • Critical aortic or pulmonic stenosis • TOF with moderate, or severe PS (ductal dependent) • TOF with pulmonary atresia • Pulmonary atresia with intact ventricular septum
CLASS IIIa	Cardiovascular anomaly or disease of important severity with the possibility or likelihood of hemodynamic instability at delivery	YES	Neonatology to manage delivery in collaboration with Cardiology	UVC + UAC	• Most transposition of the great arteries (see Class IV category for exception) • HLHS with moderate atrial level restriction, or significant tricuspid regurgitation
CLASS IIIb		NO	Neonatology to manage delivery in collaboration with Cardiology		• Total anomalous pulmonary venous connection (see Class IV category for exception) • Ebstein’s anomaly or tricuspid valve dysplasia with severe tricuspid regurgitation • TOF with Absent Pulmonary Valve Leaflet Syndrome • CHD with evidence for ventricular dysfunction • CHD with evidence for significant AV valve insufficiency • Sustained tachyarrhythmia such as SVT
IMPACT (CLASS IV)	Cardiovascular anomaly or disease in which hemodynamic instability is anticipated once separated from placental circulation thus ***IMmediate Post-partum Access to Cardiac Therapy* (IMPACT)** is implemented for urgent life-saving care	YES or NO, DEPENDS ON ANOMALY	Cardiac services including cardiac anesthesiology and Fetal Heart program to manage delivery and intervention	UVC + UAC	• HLHS with highly restrictive or intact atrial septum • Fetus with complete heart block requiring pacing • Hydropic fetus with cardiovascular disease • Transposition of the great arteries, intact ventricular septum and evident prenatal restriction of the foramen ovale • Total anomalous pulmonary venous connection with concern of obstruction • Ebstein’s anomaly or severe tricuspid valve dysplasia, with suspected pulmonary hypoplasia or anticipated respiratory insufficiency • TOF with Absent Pulmonary Valve Leaflet Syndrome and anticipated respiratory insufficiency

Class II newborns have conditions that require administration of PGE infusion to maintain ductal patency for either pulmonary or systemic blood flow, benefit from umbilical arterial monitoring, yet are anticipated to be hemodynamically stable. Class III comprises newborns with cardiovascular conditions in whom hemodynamic instability at birth is possible and close monitoring especially in the first hours of life is indicated. Alertness to the possibility for instability in the first hours of life is raised for these patients and PGE may or may not be recommended based on the expected cardiac pathophysiology. Most fetuses with transposition of the great arteries with an apparently open foramen ovale on prenatal imaging fall into Class III. At our center, neonatologists and their clinical support team care for newborns in Classes I-III in a fetal resuscitation room immediately adjacent to either the labor and delivery room or the CD operating room.

In contrast, Class IV fetuses have cardiovascular conditions in which there is a greater than 50% likelihood that the newborn will develop life-threatening distress immediately following separation from the placental circulation. These Class IV neonates are therefore candidates for the IMPACT procedure. For example, a fetus with transposition of the great arteries and prenatal restriction of the foramen ovale will almost certainly require an immediate septostomy at birth. Since life-threatening cardiovascular decompensation is predicted to take place after delivery, a multidisciplinary team composed of cardiac anesthesiologists, fetal cardiologists, interventional cardiologists, cardiac surgeons, respiratory therapists, echocardiographers, and specialty procedural/surgical nurses and technicians are assembled at the time of delivery to provide immediate postnatal resuscitation and intervention with consultative support from neonatology.

### IMPACT Patient Selection

Patients with CCHD who may require immediate postnatal interventions are initially identified by the Fetal Heart Program team. Candidacy is determined using evidence-based tools, expert opinion-based protocols to risk stratify fetuses with CCHD, and institutional consensus.

As a rule, consideration for the IMPACT procedure is reserved for fetuses with cardiovascular conditions in which there is a greater than 50% likelihood that the newborn will face life-threatening distress when they are separated from placental circulation. Numerous conditions may fall into this category of disease (Table 2). Each of these complex conditions has its own set of criteria defining suspicion for life-threatening concerns at birth [6, 9, 10, 12, 13, 19–25]. The current strategies of care offered at this institution are listed, realizing that there may be alternative successful approaches offered at other centers. While some cases clearly fulfill the criteria for requiring urgent newborn care, others may fall into zones of uncertainty. In such cases, discussion among providers and the family is carefully undertaken to best determine the risk–benefit ratio for employing an IMPACT strategy.

**Table 2:** Examples of some conditions benefitting from IMPACT procedure and planned urgent intervention at birth as per the current approach offered at The Children’s Hospital of Philadelphia

Condition	Anticipated clinical consequence	Fetal echo criteria for candidacy	Scheduled cesarean delivery & planned urgent intervention at birth
Hypoplastic left heart syndrome with intact or highly restrictive atrial septum	Severe hypoxemia at birth due to obstructed left atrial egress and possible pulmonary vasculopathy	o Intact atrial septum or small foramen ovale opening in the setting of mitral atresia or severe mitral stenosis [25] o Pulmonary vein Doppler flow pattern indicating < 3:1 anterograde:retrograde flow velocity–time integral ratio [6, 24, 25]	o Atrial septoplasty via transcatheter procedure with stenting of the atrial septum
Total anomalous pulmonary venous return with obstruction	Severe hypoxemia at birth due to restriction of pulmonary venous return	o Infra-diaphragmatic connection of total anomalous pulmonary veins, or other forms of anomalous pulmonary venous connection in which drainage appears obstructed [23]	o Transcatheter stenting with relief of obstruction in decompressing vessel o Surgical repair of anomalous pulmonary venous connection
Transposition of the great arteries with restrictive atrial septum	Severe hypoxemia at birth due to inadequate atrial level mixing	o Appearance of a small foramen ovale, or hyper-mobile redundant septum primum tissue of atrial septum [9] o Retrograde flow in the ductus arteriosus which may herald predisposition to development of atrial level restriction [10, 22] o Maternal hyperoxygenation provocative testing demonstrating elicitation of atrial restriction [21]	o Balloon atrial septostomy
Maternal autoimmune induced complete heart block	Bradycardia with inadequate cardiac output	o Ventricular heart rate of < 50 bpm [12, 13] o Heart rates > 50 bpm but with evidence of ventricular dysfunction or hydrops fetalis	o Isoproterenol infusion or other inotropic agents as indicated o Temporary epicardial pacing wire placement
Pulmonary arterio-venous fistula	Profound hypoxemia due to intra-pulmonary shunting	o Identification of pulmonary arterio-venous connection with continuous Doppler flow, retrograde flow in ductus arteriosus and cardiomegaly [19, 20]	o Catheter-based intervention for immediate occlusion of pulmonary arterio-venous fistula
Ebstein’s anomaly or severe tricuspid valve dysplasia	Circulatory or respiratory failure	o Hydrops or prenatal evidence of functional pulmonary atresia and pulmonary insufficiency with “circular shunt”	o Cardiorespiratory support, including possible ECMO support o Strategy for immediate closure of ductus arteriosus

The process of consideration for the IMPACT procedure follows a detailed protocol. Expectant mothers carrying a fetus with cardiovascular conditions are evaluated for delivery in the CHOP SDU based on fetal cardiologist (Fetal Heart Program) and maternal–fetal medicine review (Center for Fetal Diagnosis and Treatment-CFDT). Serial fetal echo imaging and serial counseling are typically performed every 4 weeks which allows for monitoring of the cardiac condition for any changes in the progression of disease. All fetuses with a diagnosis of a cardiovascular condition are assigned a classification delivery code no later than 34 weeks of gestation. Expectant mothers carrying a Class IV fetus for whom an IMPACT procedure is determined to be of benefit undergo counseling concerning the benefits and risks of the procedure for the mother and newborn. Families meet with a dedicated prenatal-focused clinical psychologist in addition to social workers to help with planning the logistics for potential relocation close to the hospital once the date for the IMPACT procedure is determined. The strategy of newborn care is discussed with the postnatal care team including cardiac anesthesiologists, cardiac interventionalists, and cardiac surgeons who will be coordinating the transition of care and orchestrating the immediate newborn intervention. The strategic plan for evaluation and intervention is discussed with the family.

Any fetal candidate for the IMPACT procedure is at risk for death during the peripartum period, the subsequent resuscitation, and any cardiac interventions that are performed. The potential role of extracorporeal membrane oxygenation (ECMO) support for newborns who would be ECMO candidates is therefore included in the IMPACT procedure counseling. In addition, engagement with the palliative care team is arranged so that the family can be familiar with the options and process of discontinuing aggressive interventional care if circumstances for consideration of such arise either before, during, or shortly after birth. Palliative care consultation does not imply limiting plans for proposed intervention(s) but is a means for families to appreciate all options available to them at various steps along the plan of care. The introduction of nonintervention as a potential option in advance can help avoid rushed and stressed decision-making discussions on the day of the IMPACT procedure and may also help families understand that nonintervention is not synonymous with failure. Parents are also offered the option to decline participation in the IMPACT strategy, should they choose to do so. For families who desire their newborn to be blessed or baptized, involvement of clergy or other faith-based providers during prenatal counseling and at the time of birth can be indicated. Religious involvement can be a powerful means of family support at a time of tremendous stress and is integrated into the care plan.

Decisions concerning the application of the IMPACT strategy are made after careful evaluation of the health of both the fetus and pregnant patient with a definitive decision ideally occurring prior to 34 weeks gestation. The ideal gestational age for delivery and IMPACT procedure is 37–39 weeks, with modifications based on the health of the fetus and pregnant patient.

### Coordination and Orchestration of the IMPACT Procedure

The IMPACT procedure demands a dedicated collaborative partnership among multiple disciplines including fetal cardiologists, obstetric and maternal–fetal medicine specialists, cardiothoracic and SDU anesthesiologists, neonatologists, interventional cardiologists, cardiac surgeons, echocardiographers, procedural nursing team, palliative care team, respiratory therapists, anesthesia technicians, social workers, and clergy, if applicable. We share the details of our approach with specific roles for each team and their participation in the coordination of this highly orchestrated endeavor.

### Preprocedural Planning and Coordination

The proposed date is communicated with all members of the team with the final date dictated by clinical factors and operator and venue availabilities. The two adjacent venues and teams, one for the pregnant patient and one for the newborn, are booked. Orders are placed in the electronic health record (EHR) for IMPACT by the primary fetal cardiologist for cardiac aspects of the unborn fetus and by the Center for Fetal Diagnosis and Treatment (CFDT) team members for the maternal CD. An IMPACT procedure email notification is sent to Cardiac Center staff outlining the patient information, indication, anticipated neonatal interventions, and proposed date.

All preprocedural orders (e.g., medications, interventional procedural orders, and blood requisition) are placed through a medical record number for the unborn baby created in the EHR that is linked to the mother’s electronic records and gets converted to the newborn’s electronic record at birth. This system allows an IMPACT procedure to be booked under the “SDU unborn” medical record number on the proposed date of the CD. This enables care coordination to begin even before the neonate is born. It also allows cardiac anesthesiologists to start an anesthesia record for vital signs and procedure documentation before the infant is officially admitted to the hospital. When the infant is officially admitted to the hospital, the medical record number stays the same but the “SDU unborn” designation is removed. Preprocedural orders and the anesthesia record remain linked to the chart during this process. Post-delivery studies are directly linked to the newborn’s chart.

The FHP prenatal cardiology team serves as the point of contact for the expectant mother. FHP nurse coordinators arrange for the family to meet with members of the other disciplines that will be involved in the care of the neonate. Serial fetal echocardiograms are performed at an interval dictated by the clinical course, and the results are communicated to the procedural and cardiac anesthesiology teams on a frequent basis.

All expectant mothers with planned delivery in the SDU (whether IMPACT delivery or not) are followed by a dedicated group of maternal–fetal medicine (MFM) specialists. In collaboration with the FHP team, these specialists conduct regular fetal assessments in the CFDT prenatal clinic, conveniently synchronized with their fetal cardiology appointments. Maternal risk factors are assessed, and obstetrical care plans are implemented. This integrated approach ensures enhanced accessibility and improved patient experience, enabling seamless management of obstetric care in collaboration with the fetal heart team. The maternal–fetal medicine specialists also coordinate the involvement of other specialties and support staff during these scheduled prenatal visits, with possible involvement of neonatology, genetics, clinical psychology, social work, palliative care, clergy, and music therapy. The pregnant patient also meets with one of the SDU anesthesiologists to review the maternal history, including possible additional records or workup for health conditions pertinent to the anesthesia plan, and to discuss the likely use of neuraxial technique for the CD.

The cardiothoracic anesthesia team serves an integral role in the management of newborns who need IMPACT procedures and leads the coordination of services at the time of delivery. In the prenatal period, a cardiothoracic anesthesiologist meets with the expectant parent(s) for counseling and to obtain consent for vascular access placement and administration of anesthesia to the neonate. Several steps are taken weeks prior to the procedure which are listed in Table 3. On the day of the procedure, this team receives the newborn from the obstetrical delivery team and leads the neonatal resuscitation efforts in the multifunctional procedural suite. A series of steps that go into the preparation for and the conduct of the neonatal stabilization is described in Table 4.

**Table 3:** List of events that need to occur prior to the day of the procedure

o Serial fetal cardiovascular evaluations, including counseling
o Genetic testing and/or amniocentesis if clinically appropriate
o Weekly case review at the FHP multi-disciplinary conference
o Palliative care consultation for the family
o Social work consultation and logistical planning for relocation
o Order placed by Fetal Heart Team in the EHR for the identified procedure
o Evaluation of the expectant mother by the MFM team and the Obstetric Anesthesiology team
o Obtain procedural and blood transfusion consents as soon as the fetus is deemed appropriate for IMPACT
o Discussion of the need for ECMO during IMPACT, if indicated notify the ECMO team
o Cardiac anesthesia consent for care of the neonate as soon as the fetus is deemed appropriate for IMPACT
o The cardiac anesthesia team places pharmacy/blood orders in EHR (specific order set is created for IMPACT patients in the EHR to facilitate order placement as soon as potential delivery date is identified and booked)
o Identify coverage for IMPACT during call time prior to the scheduled date of delivery
o Final fetal echocardiogram and FHP counseling within 1 week of IMPACT

**Table 4:** Cardiac anesthesia checklist of supplies and equipment

**Checklist for weeks prior to the procedure**
• Assigned IMPACT procedure cardiac anesthesia team identifies the mother’s chart, reviews fetal echo findings
• Through the mother’s EHR, access “SDU Unborn Baby” chart and place orders for medications weeks ahead of time so that the pharmacy can have the orders ahead of time. This can also be done against the baby’s information once the case is booked
• A dedicated IMPACT procedure order set has been created to accommodate both the usual neonatal medications (Vitamin K and erythromycin ointment) and the medications (prostaglandin, isoproterenol, inotropes, vasopressors, etc.), respiratory supports (inhalational nitric oxide) and blood bank (2 units of O negative packed red blood cells that have been crossed against a specimen from the mother) resources potentially required for immediate postnatal administration
**Checklist for equipment, supplies and personnel on the day of the procedure**
*Airway equipment* • Appropriate-sized endotracheal tube, video laryngoscope, nasal intubation supplies • ICU ventilator in the room in case ventilation and oxygenation is challenging with the ventilator on the anesthesia machine • Nitric oxide in the room if indicated
*Monitors* • ECG leads are secured to the baby using soft Velcro fasteners on to the extremities to have reliable contact with the skin surface given the presence of vernix caseosa • Pre- and post-ductal saturation measurements
*Medications (immediately accessible for administration by the appropriate/pre-assigned team members)* • Induction medications that are drawn up for the estimated fetal weight, including atropine, ketamine, fentanyl, and muscle relaxant • Emergency drugs – appropriate doses of epinephrine, phenylephrine, sodium bicarbonate and calcium gluconate • Intramuscular dose of Vitamin K, erythromycin ophthalmic ointment
*Additional supplies for resuscitation* • Warm water in a reservoir for cleaning the baby’s skin and for maintaining normothermia • Wash clothes to clean the baby • Supplies for taking footprints • Identification bracelets for baby • Lab requisitions and sample tubes for type and cross for the baby once delivered • Neonatal metabolic testing forms—must be obtained prior to blood product transfusion
*Infusions prepared* • Infusions of epinephrine and dopamine are primed and programmed on IV pumps • Prostaglandin infusion primed at 0.01–0.02 mcg/kg/min if indicated • Isoproterenol primed and programmed if indicated • Dextrose containing IV fluids (e.g., D10W or D10 LR) by infusion or on a pump • Arterial line transducer
*Vascular access supplies* OR nursing team prepares two vascular trays, each to be used separately by the attending cardiac anesthesiologists. The contents of the vascular access tray are listed below Tray 1 • 5 Fr single lumen umbilical lines X 2 • 5 Fr double lumen umbilical line X 1 • 3.5F single lumen umbilical line X1 • 3.5F double lumen umbilical line X1 • T connector and syringes for each line • Clave or sterile end for double lumen catheter Tray 2 • 5 Fr single lumen umbilical lines X 2 • 5 Fr double lumen umbilical line X 1 • 3.5F single lumen umbilical line X1 • 3.5F double lumen umbilical line X1 • T connector and syringes for each line • Clave or sterile end for double lumen catheter • 3 sterile blood gas syringes • 2 empty 10 mL syringes for blood sampling/blood type and cross

The cardiac interventional team is kept up to date on the progress of the prenatal course by the FHP team. On the day of the IMPACT procedure, the cardiac interventional team completes a huddle with the IMPACT team to discuss the assessment, planned intervention, potential pitfalls of the planned procedure, and contingency plans.

If the neonate is likely to need an immediate surgical intervention following birth (e.g., placement of temporary epicardial pacing leads in a baby with hemodynamically significant complete heart block or confirmed obstructive total anomalous pulmonary venous connection), the cardiothoracic surgical team (surgeon/nursing/perfusion) huddles and is prepared for an immediate surgical procedure.

Surgical and interventional nursing teams are kept up to date on the status of the mother with regular communication from the FHP team. The nursing and technician teams are an essential part of the neonatal resuscitation, initial stabilization, and the procedure itself.

### Day of IMPACT Procedure

**Fig. 1:**
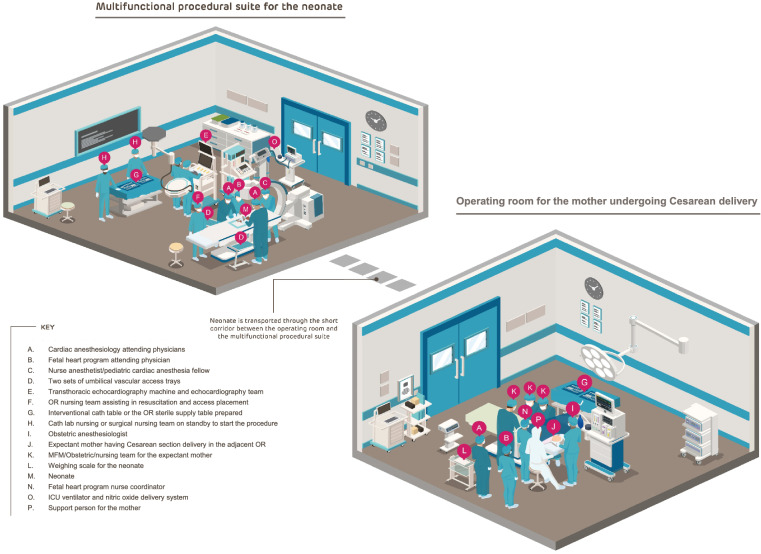
Comprehensive visual plan for optimizing staff, room, and equipment organization in the maternal Cesarean delivery operating room and the multifunctional neonatal procedural suite

The IMPACT CD is planned as the first case on a weekday in one of the operating rooms proximate to the resuscitation/intervention venue. This resuscitation/intervention venue is a multifunctional procedural suite (such as a “Hybrid room”) which has the functionality to carry out both cardiac surgical procedures and cardiac catheterization interventions. Figure 1 depicts the setup of the maternal CD suite and the multifunctional procedural suite for the neonate along with the organization of the various team members and types of equipment needed for maternal care and neonatal resuscitation and intervention. Maternal anesthesia is provided by a dedicated SDU anesthesia team. The FHP fetal cardiologist and nurse coordinator are in attendance.

After the patient receives neuraxial anesthesia, the nurse coordinator ensures that the mother’s partner or companion is positioned to provide maternal support. Pictures of the newborn immediately after delivery are taken by either the support person or the SDU team in the operating room. The neonatal procedural venue is set up by the cardiac anesthesia team (see Table 4), the team for the proposed intervention, and the cardiologists performing the post-natal echocardiogram. The neonate is received from the obstetrician by one of the attending cardiac anesthesiologists and immediately weighed and carried to the procedural venue.

A dedicated, specifically trained cardiac anesthesiology team leads the neonatal resuscitation, performance of newborn care, and expeditiously prepares the neonate for the proposed intervention. At our institution, all IMPACT procedures are staffed by two attending cardiac anesthesiologists who perform and supervise ongoing resuscitation of the newborn, including obtaining umbilical vascular access. A third anesthesia provider is assigned to every IMPACT case, who is either an advanced cardiothoracic anesthesiology fellow or a senior cardiac certified registered nurse anesthetist (CRNA). This person is primarily responsible for airway management during the resuscitation.

Initial assessment and stabilization include placement of ECG leads (pulse oximetry, and umbilical arterial and venous catheters. Neonatologists are available to offer consultative services. Once rapid umbilical vascular access is secured, the FHP cardiology team performs a clinical assessment and targeted transthoracic echocardiogram to assess pertinent features and the associated transitional changes in the immediate postnatal period. Intramuscular Vitamin K is administered, erythromycin ophthalmic ointment is applied, and a blood specimen is sent to the blood bank for blood typing and crossmatching. At our institution, a non-crossed matched, irradiated O-negative blood unit is issued for use until a crossmatching of the sample is completed. Following crossmatching, the packed red blood cell unit is made available that is fresh and irradiated. Additionally, a blood specimen for mandatory neonatal screening is obtained at the time of the first blood gas to avoid contamination should transfusion be required. Apgar scoring and newborn footprints are also completed as part of the delivery process.

Based on the immediate postnatal physiology and findings on the targeted echocardiogram, a multidisciplinary team decision is made to place an advanced airway which is often a nasal endotracheal tube. Following the initial resuscitation, additional echocardiographic assessment can be performed to acquire further anatomical and physiological assessment. Imaging, physical exam, hemodynamics, and blood gas data are integrated, and a discussion among all team members about whether to proceed with an intervention, pause and obtain additional data, or continue to observe without immediate intervention with transfer to the CICU ensues. If the decision is made to intervene, the indicated procedure is performed. The family is updated on the neonate’s status by the fetal cardiologist and FHP nurse coordinator. On completion of the procedure, the neonate is transported to the CICU. If the baby is stable, the team may first bring the patient to the SDU for the parents/family to see the baby. A sign-out is given by all members of the IMPACT team to the CICU care team.

### The Fetus May Have Other Plans: Need for IMPACT Procedure on Other than the Scheduled Date

Once an IMPACT procedure is scheduled and the baby meets viability criteria, the team commits to providing opportunity for IMPACT care around-the-clock in case of indications for earlier delivery. Whenever a fetus needing an IMPACT procedure is identified, a separate call roster is created within the cardiothoracic anesthesiology group to have a second cardiac anesthesiologist on call, through the projected date of delivery to ensure the availability of a second attending cardiac anesthesiologist if the IMPACT delivery were to take place during call time. If the expectant mother presents in preterm labor prior to the scheduled IMPACT date, the on-call MFM will contact the FHP cardiologist on-call to alert them of the need for the IMPACT fetus requiring urgent or emergent delivery during call hours. The FHP cardiologist will notify the cardiac anesthesiologist on call as well as the interventional cardiologist and/or cardiothoracic surgeon to identify the timing of delivery and to confirm that both the OR and procedural suites are available. If either is in use, the team will determine appropriate alternate venues. The cardiac anesthesiologist will notify the 2nd cardiac anesthesia attending and the cardiac anesthesia fellow or CRNA, as well as the ancillary staff. The cardiac interventionalist and or/surgeon will notify their respective teams, including the perfusion team when appropriate.

## Conclusion

The IMPACT process is a multidisciplinary, collaborative, and highly coordinated clinical service designed for expectant mothers and fetuses with CCHD with a high likelihood of neonatal instability immediately after birth. By incorporating prenatal consultations with parents and involving all relevant services from delivery to postnatal care, this process ensures comprehensive and immediate care for the newborn. The planned CD in an operating room with direct access to a multifunctional procedural suite facilitates rapid cardiac evaluation and the initiation of potentially life-saving interventional treatments. This review highlights the details of this unique procedure and multidisciplinary orchestration at one institution.

## Data Availability

No datasets were generated or analysed during the current study.
