# Correction: Transitioning from the Safety of the Womb to the Outside World for Neonates with Life-Threatening Cardiovascular Conditions: The *IMmediate Postpartum Access to Cardiac Therapy* (IMPACT) Procedure

**DOI:** 10.1007/s00246-025-03822-8

**Published:** 2025-03-18

**Authors:** Asif Padiyath, Jennifer M. Lynch, Lisa M. Montenegro, Susan C. Nicolson, Olivia Nelson, Anita L. Szwast, Amanda J. Shillingford, Christine B. Falkensammer, Jill J. Savla, Julie Moldenhauer, Nahla Khalek, Jack Rychik

**Affiliations:** 1 Division of Cardiothoracic Anesthesiology, Department of Anesthesiology and Critical Care Medicine, Children’s Hospital of Philadelphia, Philadelphia, PA USA; 2 Division of Cardiology, Department of Pediatrics, Children’s Hospital of Philadelphia, Philadelphia, PA USA; 3 Department of Anesthesiology and Critical Care, Perelman School of Medicine at the, University of Pennsylvania, Philadelphia, PA USA; 4 Department of Bioengineering, School of Engineering and Applied Science, University of Pennsylvania, Philadelphia, PA USA; 5 Perelman School of Medicine at the University of Pennsylvania, University of Pennsylvania Health System, Philadelphia, PA USA; 6 Section of Fetal Anesthesia, Division of General Anesthesiology, Department of Anesthesiology and Critical Care Medicine, Children’s Hospital of Philadelphia, Philadelphia, PA USA; 7 Fetal Heart Program, Division of Cardiology. Department of Pediatrics, Children’s Hospital of Philadelphia, Philadelphia, PA USA; 8 Department of Pediatrics, Perelman School of Medicine at the University of Pennsylvania, Philadelphia, PA USA; 9 Division of Maternal-Fetal Medicine, Richard D. Wood Jr. Center for Fetal Diagnosis and Treatment (CFDT), The Children’s Hospital of Philadelphia, Philadelphia, PA USA; 10 Department of Surgery, Perelman School of Medicine at the, University of Pennsylvania, Philadelphia, PA USA; 11 Department of Obstetrics and Gynecology, Perelman School of Medicine at the University of Pennsylvania, Philadelphia, PA USA; 12 Fontan Rehabilitation, Wellness, Activity and Resilience Development (FORWARD) Program, Division of Cardiology, Department of Pediatrics, Children’s Hospital of Philadelphia, Philadelphia, PA USA

**Correction to: Pediatric Cardiology** 10.1007/s00246-025-03775-y

In Table 1 of this article, the data in the fifth row of the first column was incorrectly stated as “class IIIa” and should have been “class IIIb”. The incorrect and the corrected versions of Table 1 are given below.

Incorrect Table:

**Table 1:** Severity classification for delivery of fetuses with cardiovascular disease at The Children’s Hospital of Philadelphia—Special Delivery Unit

	Definition	PGE?	Action	Access	Example anomalies/diseases*
CLASS Ia	Simple cardiovascular anomaly or disease, requiring delivery in the SDU but no hemodynamic instability anticipated at delivery	NO	Neonatology to manage delivery	PIV, no UAC	∙ LV/RV size discrepancy with suspicion of possible Coarctation of the aorta ∙ AV Canal, balanced ∙ TOF with mild PS (“Pink TOF”) ∙ Benign arrhythmia (non-sustained SVT, no hydrops, no ventricular dysfunction)
CLASS Ib			Neonatology to manage delivery	UVC + UAC	∙ Truncus Arteriosus ∙ LV-RV disproportion, with intent to test ductal closure for possible coarctation of aorta
CLASS II	Cardiovascular anomaly or disease of moderate severity, including ductal dependent lesions. Hemodynamic instability is possible, but unlikely and not anticipated at delivery	YES	Neonatology to manage delivery	UVC + UAC	∙ HLHS, no risk factors (open inter-atrial communication, no significant TR, good RV function) ∙ Single ventricle with critical (suspect ductal dependent) pulmonary outflow obstruction (e.g., tricuspid atresia) ∙ Single ventricle with critical (suspect ductal dependent) systemic outflow obstruction (e.g., Double-inlet LV with arch obstruction) ∙ Coarctation of the aorta ∙ Critical aortic or pulmonic stenosis ∙ TOF with moderate, or severe PS (ductal dependent) ∙ TOF with pulmonary atresia ∙ Pulmonary atresia with intact ventricular septum
CLASS IIIa	Cardiovascular anomaly or disease of important severity with the possibility or likelihood of hemodynamic instability at delivery	YES	Neonatology to manage delivery in collaboration with Cardiology	UVC + UAC	∙ Most transposition of the great arteries (see Class IV category for exception) ∙ HLHS with moderate atrial level restriction, or significant tricuspid regurgitation
CLASS IIIa		NO	Neonatology to manage delivery in collaboration with Cardiology		∙ Total anomalous pulmonary venous connection (see Class IV category for exception) ∙ Ebstein’s anomaly or tricuspid valve dysplasia with severe tricuspid regurgitation ∙ TOF with Absent Pulmonary Valve Leaflet Syndrome ∙ CHD with evidence for ventricular dysfunction ∙ CHD with evidence for significant AV valve insufficiency ∙ Sustained tachyarrhythmia such as SVT
IMPACT (CLASS IV)	Cardiovascular anomaly or disease in which hemodynamic instability is anticipated once separated from placental circulation thus ***IMmediate Post-partum Access to Cardiac Therapy* (IMPACT)** is implemented for urgent life-saving care	YES or NO, DEPENDS ON ANOMALY	Cardiac services including cardiac anesthesiology and Fetal Heart program to manage delivery and intervention	UVC + UAC	∙ HLHS with highly restrictive or intact atrial septum ∙ Fetus with complete heart block requiring pacing ∙ Hydropic fetus with cardiovascular disease ∙ Transposition of the great arteries, intact ventricular septum and evident prenatal restriction of the foramen ovale ∙ Total anomalous pulmonary venous connection with concern of obstruction ∙ Ebstein’s anomaly or severe tricuspid valve dysplasia, with suspected pulmonary hypoplasia or anticipated respiratory insufficiency ∙ TOF with Absent Pulmonary Valve Leaflet Syndrome and anticipated respiratory insufficiency

Corrected Table:

**Table 1:** Severity classification for delivery of fetuses with cardiovascular disease at The Children’s Hospital of Philadelphia—Special Delivery Unit

	Definition	PGE?	Action	Access	Example anomalies/diseases*
CLASS Ia	Simple cardiovascular anomaly or disease, requiring delivery in the SDU but no hemodynamic instability anticipated at delivery	NO	Neonatology to manage delivery	PIV, no UAC	∙ LV/RV size discrepancy with suspicion of possible Coarctation of the aorta ∙ AV Canal, balanced ∙ TOF with mild PS (“Pink TOF”) ∙ Benign arrhythmia (non-sustained SVT, no hydrops, no ventricular dysfunction)
CLASS Ib			Neonatology to manage delivery	UVC + UAC	∙ Truncus Arteriosus ∙ LV-RV disproportion, with intent to test ductal closure for possible coarctation of aorta
CLASS II	Cardiovascular anomaly or disease of moderate severity, including ductal dependent lesions. Hemodynamic instability is possible, but unlikely and not anticipated at delivery	YES	Neonatology to manage delivery	UVC + UAC	∙ HLHS, no risk factors (open inter-atrial communication, no significant TR, good RV function) ∙ Single ventricle with critical (suspect ductal dependent) pulmonary outflow obstruction (e.g., tricuspid atresia) ∙ Single ventricle with critical (suspect ductal dependent) systemic outflow obstruction (e.g., Double-inlet LV with arch obstruction) ∙ Coarctation of the aorta ∙ Critical aortic or pulmonic stenosis ∙ TOF with moderate, or severe PS (ductal dependent) ∙ TOF with pulmonary atresia ∙ Pulmonary atresia with intact ventricular septum
CLASS IIIa	Cardiovascular anomaly or disease of important severity with the possibility or likelihood of hemodynamic instability at delivery	YES	Neonatology to manage delivery in collaboration with Cardiology	UVC + UAC	∙ Most transposition of the great arteries (see Class IV category for exception) ∙ HLHS with moderate atrial level restriction, or significant tricuspid regurgitation
CLASS IIIb		NO	Neonatology to manage delivery in collaboration with Cardiology		∙ Total anomalous pulmonary venous connection (see Class IV category for exception) ∙ Ebstein’s anomaly or tricuspid valve dysplasia with severe tricuspid regurgitation ∙ TOF with Absent Pulmonary Valve Leaflet Syndrome ∙ CHD with evidence for ventricular dysfunction ∙ CHD with evidence for significant AV valve insufficiency ∙ Sustained tachyarrhythmia such as SVT
IMPACT (CLASS IV)	Cardiovascular anomaly or disease in which hemodynamic instability is anticipated once separated from placental circulation thus ***IMmediate Post-partum Access to Cardiac Therapy* (IMPACT)** is implemented for urgent life-saving care	YES or NO, DEPENDS ON ANOMALY	Cardiac services including cardiac anesthesiology and Fetal Heart program to manage delivery and intervention	UVC + UAC	∙ HLHS with highly restrictive or intact atrial septum ∙ Fetus with complete heart block requiring pacing ∙ Hydropic fetus with cardiovascular disease ∙ Transposition of the great arteries, intact ventricular septum and evident prenatal restriction of the foramen ovale ∙ Total anomalous pulmonary venous connection with concern of obstruction ∙ Ebstein’s anomaly or severe tricuspid valve dysplasia, with suspected pulmonary hypoplasia or anticipated respiratory insufficiency ∙ TOF with Absent Pulmonary Valve Leaflet Syndrome and anticipated respiratory insufficiency

The original article has been corrected.

